# Identification and Verification of Novel Biomarkers Involving Rheumatoid Arthritis with Multimachine Learning Algorithms: An In Silicon and In Vivo Study

**DOI:** 10.1155/2024/3188216

**Published:** 2024-02-14

**Authors:** Fucun Liu, Juelan Ye, Shouli Wang, Yang Li, Yuhang Yang, Jianru Xiao, Aimin Jiang, Xuhua Lu, Yunli Zhu

**Affiliations:** ^1^Department of Orthopedics, Changzheng Hospital, Naval Medical University, Shanghai, China; ^2^Wuxi School of Medicine, Jiangnan University, Wuxi, Jiangsu, China; ^3^Spinal Tumor Center, Changzheng Hospital, Naval Medical University, Shanghai, China; ^4^Orthopedics Research Center, Taizhou Hospital of Zhejiang Province Affiliated to Wenzhou Medical University, Zhejiang, China; ^5^Department of Urology, Changhai Hospital, Naval Medical University, Shanghai, China

## Abstract

**Background:**

Rheumatoid arthritis (RA) remains one of the most prevalent chronic joint diseases. However, due to the heterogeneity among RA patients, there are still no robust diagnostic and therapeutic biomarkers for the diagnosis and treatment of RA.

**Methods:**

We retrieved RA-related and pan-cancer information datasets from the Gene Expression Omnibus and The Cancer Genome Atlas databases, respectively. Six gene expression profiles and corresponding clinical information of GSE12021, GSE29746, GSE55235, GSE55457, GSE77298, and GSE89408 were adopted to perform differential expression gene analysis, enrichment, and immune component difference analyses of RA. Four machine learning algorithms, including LASSO, RF, XGBoost, and SVM, were used to identify RA-related biomarkers. Unsupervised cluster analysis was also used to decipher the heterogeneity of RA. A four-signature-based nomogram was constructed and verified to specifically diagnose RA and osteoarthritis (OA) from normal tissues. Consequently, RA-HFLS cell was utilized to investigate the biological role of *CRTAM* in RA. In addition, comparisons of diagnostic efficacy and biological roles among *CRTAM* and other classic biomarkers of RA were also performed.

**Results:**

Immune and stromal components were highly enriched in RA. Chemokine- and Th cell-related signatures were significantly activated in RA tissues. Four promising and novel biomarkers, including *CRTAM*, *PTTG1IP*, *ITGB2*, and *MMP13*, were identified and verified, which could be treated as novel treatment and diagnostic targets for RA. Nomograms based on the four signatures might aid in distinguishing and diagnosing RA, which reached a satisfactory performance in both training (AUC = 0.894) and testing (AUC = 0.843) cohorts. Two distinct subtypes of RA patients were identified, which further verified that these four signatures might be involved in the immune infiltration process. Furthermore, knockdown of *CRTAM* could significantly suppress the proliferation and invasion ability of RA cell line and thus could be treated as a novel therapeutic target. CRTAM owned a great diagnostic performance for RA than previous biomarkers including *MMP3*, *S100A8*, *S100A9*, *IL6*, *COMP*, *LAG3*, and *ENTPD1*. Mechanically, CRTAM could also be involved in the progression through immune dysfunction, fatty acid metabolism, and genomic instability across several cancer subtypes.

**Conclusion:**

*CRTAM*, *PTTG1IP*, *ITGB2*, and *MMP13* were highly expressed in RA tissues and might function as pivotal diagnostic and treatment targets by deteriorating the immune dysfunction state. In addition, *CRTAM* might fuel cancer progression through immune signals, especially among RA patients.

## 1. Introduction

As an often-debilitating systemic autoimmune disease, rheumatoid arthritis (RA) is one of the most common autoimmune diseases [1–3]. RA is an autoimmune disease characterized by chronic inflammation and affects approximately 0.5%–1% of the world's population, most of whom are women [4]. There are four main signs and symptoms of RA: hyperplastic synovium, persistent synovitis, injuries to joints and cartilage, etc. [5, 6]. There are several causes of RA, including environmental factors and genetic factors [7]. To date, relatively little is known about the causes and mechanisms of RA, not to mention robust and reproductive biomarkers. Thus, it is imperative to develop novel RA-related targets for better diagnosis and management of RA patients, which is the focus of current studies.

The development of high-throughput sequencing technology has resulted in the emergence of an increasing number of RA-related datasets [8–11]. Although several RA biomarker-related studies have emerged, most of the research was based on a single RA dataset and applied one type of machine learning algorithm, which might cause data bias and unreliability of the results. Using biomarker profiling with machine learning allows for evidence-based clinical management [12]. A growing number of interest studies have emerged with the use of machine learning (ML) for RA biomarker development. Zhao et al. [13] downloaded three Gene Expression Omnibus (GEO) datasets with RF, SVM, and Lasso algorithms, which suggested that *BTN3A2*, *CYFIP2*, *ST8SIA1*, and *TYMS* could be adopted for RA diagnosis. Zhou et al. [14] applied the Wilcoxon test and LASSO regression from five RA microarray datasets and found that *CCL5*, *CXCR4*, *GZMA*, and *CD8A* could be treated as diagnostic biomarkers for RA. A study from Jiang et al. [15] also reported that three hub genes, *CKS2*, *CSTA*, and *LY96*, had high diagnostic values for RA after applying weighted gene coexpression network analysis (WGCNA) and LASSO regression. Even though those findings might help better understand RA and provide new perspectives on RA systematic diagnosis and therapy, the sample size and limited algorithm numbers might weaken the reliability and robustness to some extent. More advanced algorithms, such as Xgboost, could serve as an effective approach to identify novel RA-related biomarkers. In addition, the presence of sustained inflammation is one of the hallmarks of tumor promotion, which correlates with the poor prognosis of multiple types of cancer. Whether RA can increase tumor susceptibility and progression remains largely unlearned.

In this study, we aimed to integrate all public RA datasets containing more than 10 samples to discover and verify promising RA biomarkers. We adopted six corresponding cohorts and four mainstream and advancing machine learning algorithms to extract RA-related biomarkers. The differences in biological processes and immune components were investigated. We illustrated the inner association of those biomarkers and the immune heterogeneity of RA. Moreover, the potential impact of RA on cancer progression was also investigated in our work.

## 2. Materials and Methods

### 2.1. Data Collection and Processing

Six public RA datasets from GEO were downloaded and processed, which consisted of GSE12021 (*N* = 21, normal tissue = 12, RA tissues = 9), GSE29746 (*N* = 35, normal tissue = 9, RA tissues = 26), GSE55235 (*N* = 35, normal tissue = 9, RA tissues = 16), GSE55457 (*N* = 20, normal tissue = 9, RA tissues = 11), GSE77298 (*N* = 23, normal tissue = 7, RA tissues = 16), and GSE89408 (*N* = 180, normal tissue = 28, RA tissues = 152) [16–21]. The former five datasets were integrated, and the batch was removed to construct a training cohort, while GSE89408 was treated as an independent test cohort. GSE55235, GSE55457, GSE82107, GSE12021, and GSE1919 (*N* = 180, normal tissue = 5, OA tissues = 152, RA tissues = 5) were adopted to test whether biomarkers could distinguish OA and RA from normal tissues [22]. The baseline information of six datasets was summarized in *Supplementary 1*. In addition, five fresh unpaired patients collected from joint surgery, derived normal and RA tissues from Changzheng Hospital, were collected for different expression validation. For RA cohorts from public databases, institutional review board approval and informed consent were not needed. In addition, pan-cancer information, including 33 types of cancers, was retrieved from the GDC The Cancer Genome Atlas portal to identify the role across various cancers [23].

### 2.2. Batch Effect Removal

To remove the batch effect derived from study design, sequence platform, and technological replication, we filtered only normal and RA tissue expression matrices and clinical characteristics from five cohorts, including GSE12021, GSE29746, GSE55235, GSE55457, and GSE77298. Then, the batch effect was removed with the use of the default function from the package sva. Principal component analysis (PCA) was adopted to visualize the efficacy of batch removal.

### 2.3. Differential Expression and Enrichment Analysis

We used the limma package to identify differentially expressed genes (DEGs) between normal and RA tissues in the merged expression matrix. The threshold to filter significantly DEGs was as follows: *p* value < 0.05 and abstract log fold-change >1.2. Then, the packages Clusterprofiler, ggpplot2, and enrichplot were adopted to further perform enrichment analysis of DEGs [24]. Gene ontology (GO), Kyoto Encyclopedia of Genes and Genomes (KEGG) and gene set enrichment analysis (GSEA) were performed to better understand the biological role of those DEGs. The significantly different enrichment terms or pathways were identified by the threshold *p* value < 0.05 and *q* value < 0.05.

### 2.4. Identification and Verification of RA-Related Biomarkers

After identifying DEGs from the combined expression profile from five GEO datasets, we next sought to identify the most relevant RA-derived biomarkers. We adopted four machine-learning algorithms, including least absolute shrinkage and selection operator (LASSO) logistic, random forest (RF), eXtreme Gradient Boosting (Xgboost), and support vector machine (SVM), to select reliable markers to distinguish between normal and RA tissues. LASSO logistics is a model for classification problems that uses L1 regularization for feature selection and parameter reduction (the detailed parameters were as follows: alpha = 1, maximum number of iterations = 5,000, tol = 1e − 4). Random forest works as an integrated learning algorithm that combines multiple decision trees to perform classification or regression (the detailed parameters were as follows: n_estimators = 80, criterion = gini, min_samples_split = 2, min_samples_leaf = 1). XGBoost is a gradient boosting tree algorithm that performs classification or regression by integrating multiple decision trees in a boosting framework (the detailed parameters were as follows: *n*_estimators = 200, max_depth = 6, reg_alpha = 0, colsample_bytree = 1). SVM functions as a classic classification and regression algorithm and a well-labeled input were required, and the combined datasets were divided into training and test cohorts with the parameter of fivefold to perform cross validation (the detailed parameters were as follows: tol = 1e − 3, max_iter = −1). Biomarkers with ROC value large than 0.75 to distinguish RA from normal tissues in each machine learning were selected, and the interaction of biomarkers with high accuracy were finally selected to constructed diagnostic model. The receiver operating characteristic (ROC) curve was adopted to further evaluate the accuracy of filtered biomarkers in the training and testing cohorts with the use of the package pROC. Furthermore, biomarkers owing ROC value large than 0.75 in each machine learning algorithm were selected to construct the diagnostic model of RA.

### 2.5. Immune Component and Cell Differences between Normal and RA Tissues

The input file for immune deconvolution analysis was based on transcriptome expression matrix of RA. Two deconvolution algorithms, single sample gene set enrichment analysis (ssGSEA) and ESTIMATE, were enrolled in our work. ssGSEA and bulk sequenced-based deconvolution algorithms from R packages GSVA and estimate, respectively, were utilized to detect different immune cells and components between normal and RA tissues [25]. The ssGSEA algorithm is a method for GSEA, which differs from the traditional GSEA algorithm in that it can perform GSEA on a single sample and is suitable for small samples or single cell data. ESTIMATE algorithm is a method for estimating the proportion of stromal and immune cells in tissue. The basic idea is to use gene expression data to infer the amount of normal tissue cells in the tissue and thus indirectly the amount of stromal and immune cells. The gene sets used for ssGSEA consisted of 28 types of immune cells, including activated B cells, activated CD4 T cells, activated CD8 T cells, activated dendritic cells, CD56 bright natural killer cells, CD56 dim natural killer cells, central memory CD4 T cells, central memory CD8 T cells, effector memory CD4 T cells, effector memory CD8 T cells, eosinophils, gamma delta T cells, immature B cells, immature dendritic cells, macrophages, mast cells, MDSCs, memory B cells, monocytes, natural killer cells, natural killer T cells, neutrophils, plasmacytoid dendritic cells, regulatory T cells, T follicular helper cells, type 1 T helper cells, type 17 T helper cells, and type 2 T helper cells. Estimate algorithms contained three scores: ESTIMATE, immune score, and stromal score. The deconvolution file for ssGSEA were summarized in *Supplementary 2*. The details for estimate algorithm were as follows: among which, ESTIMATE score for each sample was calculated by solving a linear regression model by inversion using a known normal gene expression dataset as a reference. ESTIMATE score represented the amount of nondiseased cells in the sample. Then, ESTIMATE score was used to adjust the gene expression data to remove the nonlesioned component to obtain pure lesioned gene expression data and then to calculate the number of stromal cells in the sample using a collection of marker genes for the stromal cells. Finally, the gene expression data were also adjusted using ESTIMATE score and then the set of marker genes for the immune cells in question was used to calculate the number of immune cells in each sample.

### 2.6. Validation of RA-Related Biomarkers in Clinical Samples and Investigating the Role of CRTAM

Based on the protocol's instructions, we extracted total RNA from patient-derived tissues (after joint surgery in Changzheng Hospital) using TRIzol reagent (Invitrogen, Carlsbad, CA, USA). The Reverse Transcription Kit was utilized to perform qRT-PCR assays (Takara, Dalian, China). We also used the Fast Real-Time PCR 7500 System (Applied Biosystems, Foster City, CA, USA) to quantify gene expression levels. *GAPDH* was amplified and treated as the internal control. The relative quantification values for the four biomarkers were calculated by the 2^−*DDCt*^ method. The primers were as follows: *CRTAM* (forwards primer: GACGCTCACTCTAAAGTGTGTC; and reverse primer: CTTGCAGGGTTACGTTAGGCA), *PTTG1IP* (forwards primer: GTCTGGACTACCCAGTTACAAGC; and reverse primer: CGCCTCAAAGTTCACCCAA), ITGB2 (forwards primer: TGCGTCCTCTCTCAGGAGTG; and reverse primer: GGTCCATGATGTCGTCAGCC), *MMP13* (forwards primer: ACTGAGAGGCTCCGAGAAATG; and reverse primer: GAACCCCGCATCTTGGCTT), and *GAPDH* (forwards primer: ACAACTTTGGTATCGTGGAAGG; and reverse primer: GCCATCACGCCACAGTTTC). RA-related cell line, RA-HFLS, was obtained from Immocell, Inc., and cultured in DMEM medium containing 10% FBS and 1% penicillin/streptomycin. The siRNAs, including siNC and siCRTAM, were purchased from Shanghai GeneChem Co., Ltd. The interferon effect was tested by Western blotting and a-PCR. The growth curve of RA were detected via cell counting Kit-8 assay, and all procedures were performed with the reference of protocols of manufacturer. Cell cycle assays were performed by flow cytometer with the use of PI staining, which were quantified by FACSCalibur (Becon Dickinson, NY, USA). Annexin VFITC and PI staining kits were purchased from BD Biosciences in the USA for the purpose of analyzing apoptosis in RA-HFLS cells. Migration assays were carried out using Transwell chambers (Corning; catalog no. 3422). The transfected cells were seeded into the upper chamber with serum-free medium, specifically 1 × 10^4^ cells, while the bottom of the chamber contained DMEM supplemented with 20% fetal bovine serum (FBS). Following a 48 hr incubation period, the cells were fixed and stained with crystal violet. Quantification of the migrated or invaded cells was performed by counting the number of cells in three random fields at a magnification of ×100 using an inverted light microscope from Leica, model DMI3000. Additionally, the impact of CRTAM on RA-HFLS cell proliferation was assessed through a colony formation assay. In addition, we applied the Spearman correlation to investigate the potential biological role of CRTAM in RA. The correlation index between CRTAM and remained m RNA signatures of combined RA transcriptome were calculated. Then, we ranked all genes according to correlated index to perform KEGG and GAVA analysis, and the top 500 most relevant genes were chosen to perform GO analysis.

### 2.7. Identification of Different Subtypes of RA

To further reveal the heterogeneity of RA, we performed an unsupervised cluster analysis based on the four novel biomarkers with the use of the ConsensusClusterPlus package [26]. The optimal cluster number was identified based on the cumulative distribution function (CDF) curve and PCA algorithm. Then, estimates and immune differences were also compared between subtypes.

### 2.8. Statistical Analysis

All data processing, statistical tests, and result visualization were completed in R software (version 4.1.2). We used the *t*-test or Kruskal‒Wallis test to compare quantitative variables with the ggpubr package. Kaplan‒Meier plotter was adopted to analyze the prognostic impact of CRTAM on different cancers. The variable's correlation index was calculated through Pearson and Spearman correlation tests. Correlation analysis and genemania website were applied to investigate the biological roles of *CRTAM* and other publica biomarkers including *MMP3*, *S100A8*, *S100A9*, *IL6*, *COMP*, *LAG3*, and *ENTPD1*. All validation experiments were repeated three times independently. All two-sided *p* values (<0.05) were considered statistically significant.

## 3. Results

### 3.1. Extracting Hub Signatures of RA Tissues

**Figure 1:**
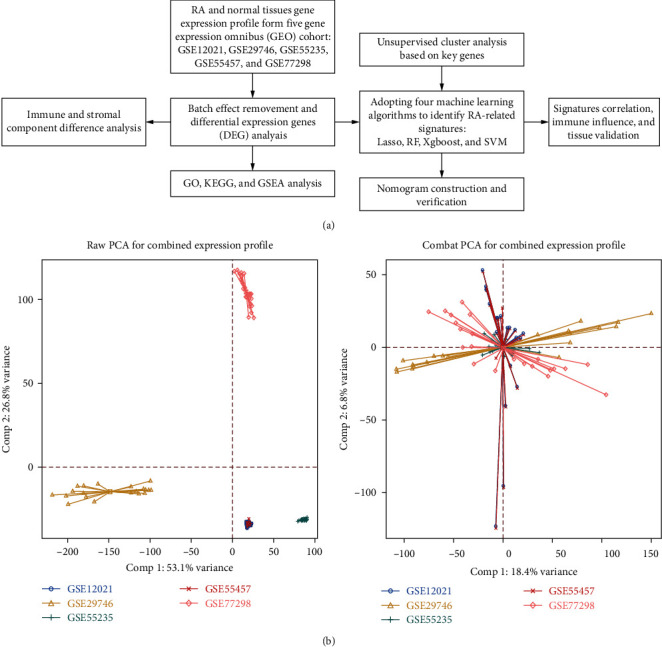
Workflow and batch removal. (a) Overall workflow of this study. (b) PCA plot illustrating the efficiency of batch effect removal (left: before; right: after batch effect removal).

The study dataset processing and whole workflow are briefly summarized in Figure 1(a). To construct a more comprehensive dataset containing balanced ratios of normal and RA tissue samples, we selected GSE12021, GSE29746, GSE55235, GSE55457, and GSE77298 to build a novel cohort for further DEG and machine learning processes. The batch effect was satisfactorily removed, and datasets were integrated, which included 107 samples (RA: 60, normal tissues: 47) (Figure 1(b)).

**Figure 2:**
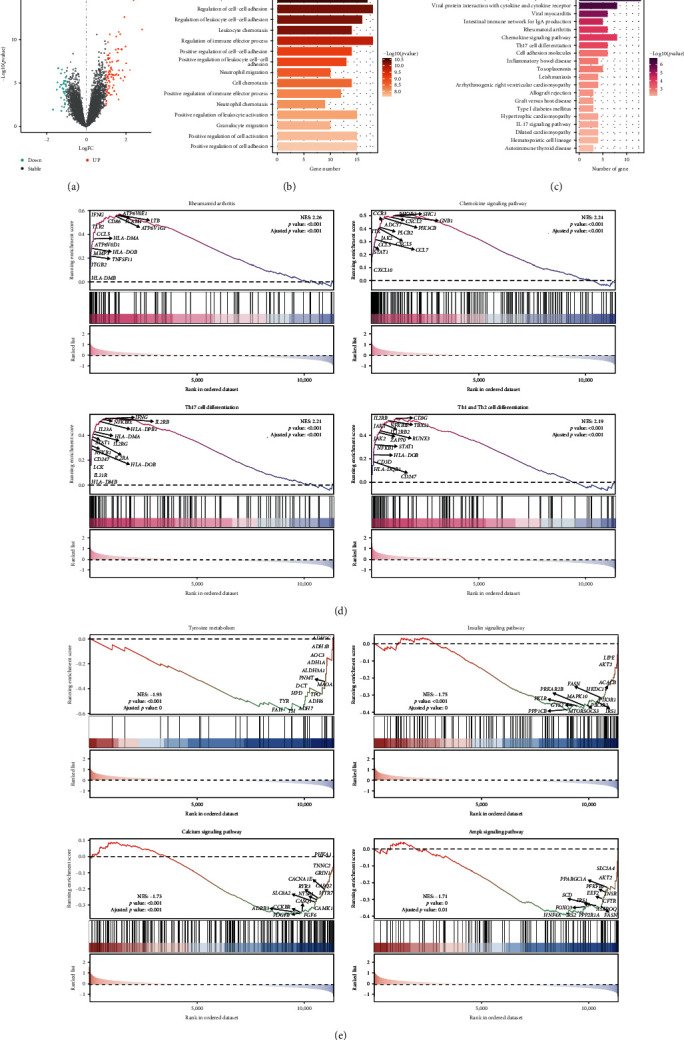
Differentially expressed signatures between RA and normal tissues. (a) Volcano plot of differentially expressed genes. Red represents upregulated genes; blue represents downregulated genes. (b, c) Bar plot of BP and KEGG pathway enrichment analysis of all DEGs. (d, e) GSEA indicates the most activated and inhibited pathways in RA compared with normal tissues.

Differential expression analysis further identified 117 DEGs (84 upregulated and 33 downregulated genes) in RA tissues compared with normal tissues (Figure 2(a)). In addition to cell‒cell regulation, those DEGs were also annotated in regulation of immune effector process and neutrophil migration in biological process; MHC protein complex, immunological synapse, and dystrophin-associated glycoprotein complex in cellular component; serine hydrolase activity, active ion transmembrane transporter activity, and anion transmembrane transporter activity in molecular function (Figure 2(b), *Supplementary 3*). In addition, these DEGs were enriched in cytokine−cytokine receptor interactions and protein interactions with cytokines and cytokine receptor-related processes in KEGG analysis (Figure 2(c)). Furthermore, through GSEA, we found that, in addition to the classic RA pathway, chemokine signaling and Th17, Th1, and Th2 cell differentiation were also activated in the progression of RA, while tyrosine metabolism, insulin signaling, calcium, and AMPK signaling pathways were downregulated in RA (Figures 2(d) and 2(e)).

### 3.2. Identification of RA-Related Signatures through Multimachine Learning Algorithms

**Figure 3:**
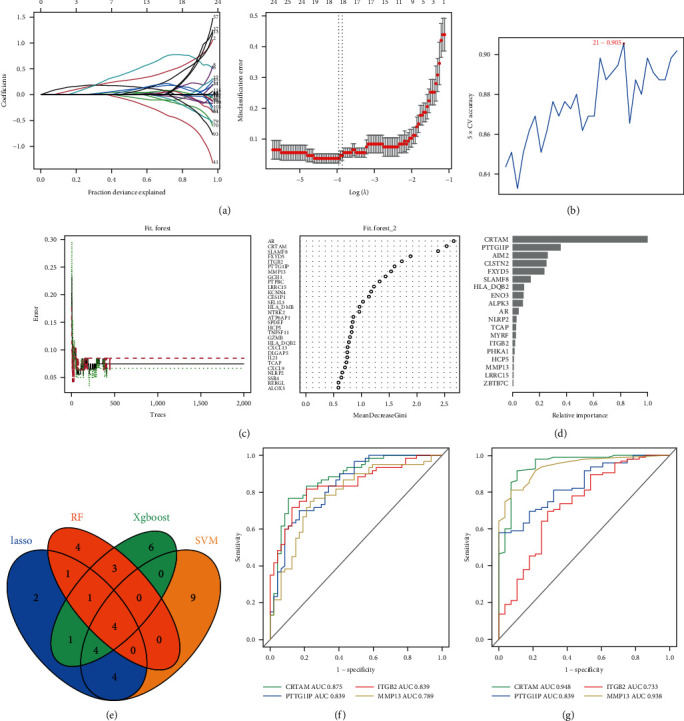
Identification and verification of RA-related signatures. (a) LASSO, (b) SVM–RFE, (c) RF, and (d) XGBoost algorithms were applied to identify RA-related biomarkers based on DEGs in the discovery cohort. (e) Intersections of features from the four machine learning algorithms in the discovery cohort. (f, g) ROC curves were used to evaluate the specificity and sensitivity of the four intersection signatures to distinguish RA and normal tissues in the discovery and validation cohorts.

In this part, we aimed to find the most relevant and promising RA-related biomarkers. We first retracted the 107 DEGs identified previously and constructed expression profiles of normal and RA tissues. Then, four algorithms, including LASSO logistic, SVM recursive feature elimination, RF algorithms, and XGBoost, were introduced to identify RA-related signatures (Figure 3(a)–3(d)). After integrating the merged signatures, four novel RA-related biomarkers (*CRTAM*, *PTTG1IP*, *ITGB2*, and *MMP13*) were identified (Figure 3(e)). We next used ROC curves to evaluate the specificity and sensitivity of those four signatures to distinguish RA and normal tissues. As Figure 3(f) indicates, all the AUC values of *CRTAM*, *PTTG1IP*, *ITGB2*, and *MMP13* were higher than 0.75, and the AUC value of *CRTAM* reached 0.875. All these findings were further validated in the GSE89408 cohort (Figure 3(g)). Our findings suggested the potential of *CRTAM*, *PTTG1IP*, *ITGB2*, and *MMP13* as promising diagnostic biomarkers for RA patients. We also decided to test whether the four signatures could distinguish OA from normal tissues, since several promising studies revealed that several targets were applicable to achieve improved and synergistic treatment efficiency between RA and OA. The AUC values from five OA datasets suggested that four signature-based predictors could also work as specific biomarkers for OA, and nearly all signatures were more highly expressed in OA tissues (*Supplementary 4*). All these findings proved that our model might be useful to distinguish RA and OA from normal tissues, respectively.

### 3.3. RA Displayed an Immune-Activated State Compared with Normal Tissues

**Figure 4:**
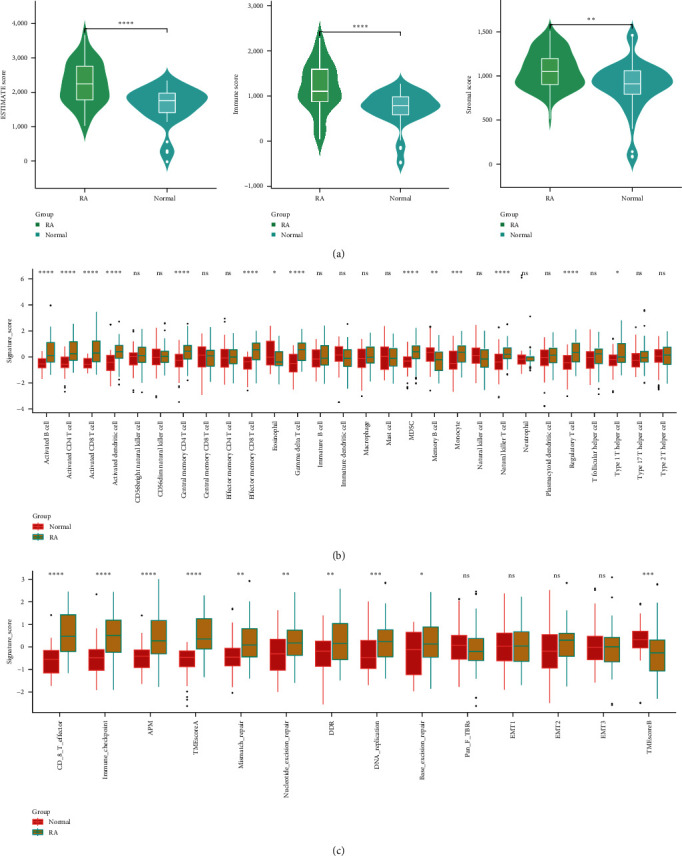
Differences in immune components and signatures between RA and normal tissues. (a) Differences in ESTIMATE score, immune score, and stromal score between RA and normal tissues. (b, c) Different infiltration degrees of 28 types of immune cells and immune signatures between RA and normal tissues.  ^*∗*^*P* < 0.05,  ^*∗∗*^*P* < 0.01,  ^*∗∗∗*^*P* < 0.001,  ^*∗∗∗∗*^*P*-value is too small, close to zero.

Previous results in this study reminded us that the immune-related signature might be involved in the pathogenesis of RA. We next compared the potential immune difference between RA and normal tissues. The estimated results indicated that RA displayed a significantly higher ESTIMATE, immune score, and stromal score than normal tissues (Figure 4(a)). We also found that most immune cells, including activated B cells, activated CD4 T cells, activated CD8 T cells, activated dendritic cells, central memory CD4 T cells, effector memory CD8 T cells, gamma delta T cells, MDSCs, monocytes, natural killer T cells, regulatory T cells, and type 1 T helper cells, were more highly enriched in RA tissue, while eosinophils and memory B cells were less infiltrated in RA tissues (Figure 4(b)). In addition, we validated such an immune hot or higher infiltrated state through another immune signature set (Figure 4(c)), which suggested that the whole immune process was activated in RA tissues.

### 3.4. Impact of Signatures on RA Immunological Infiltration and Biological Role of CRTAM

**Figure 5:**
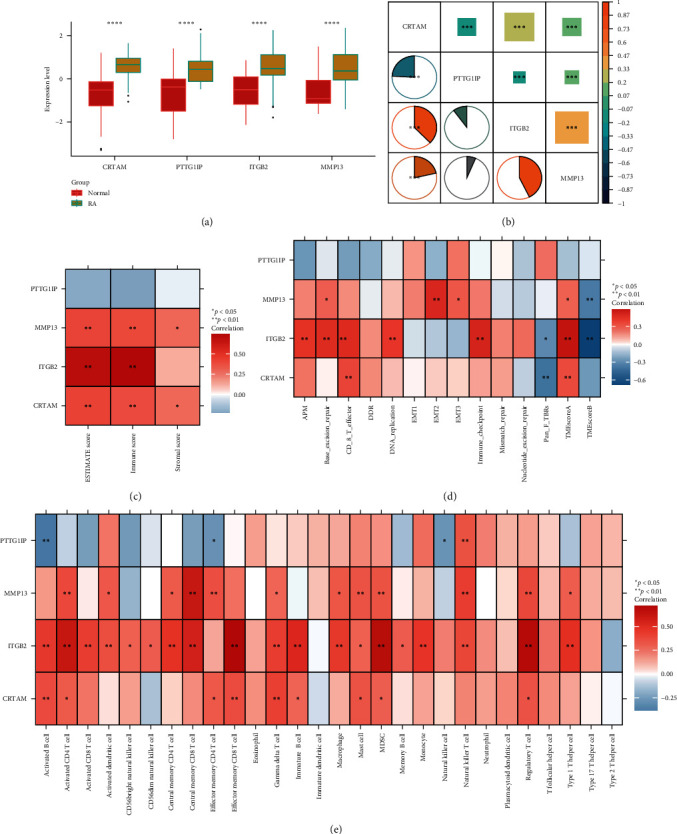
Correlation of four biomarkers and immune signatures in RA. (a) Different expression levels of four biomarkers between normal and RA tissues. (b) Spearman and Pearson correlations of four biomarkers in the RA expression matrix. (c–e) Relationship of four biomarkers and estimated related scores, immune-related signature scores, and immune cell infiltration scores in RA.  ^*∗∗∗*^*P* < 0.001,  ^*∗∗∗∗*^*P*-value is too small, close to zero.

Figure 5(a) indicates that *CRTAM*, *PTTG1IP*, *ITGB2*, and *MMP13* were more highly expressed in RA tissues, and we utilized patient-derived tissues to further validate these differences (*Supplementary 5*). Since *CRTAM* led the best discriminative power between RA and normal tissues (Figures 3(f) and 3(g)), we next aimed to investigate the detailed biological function of *CRTAM* in RA. The knockdown efficacy was verified by WB and q-PCR (*Supplementary 5*); thus, we chose the optimal siRNA of *CRTAM* to perform in vitro experiments. The proliferation ability of HFLS was significantly inhibited in *CRTAM* knockdown group (*Supplementary 5*). Furthermore, *CRTAM* knockdown could trigger HFLS cell cycle arrest in G1 phase compare with NC group (*Supplementary 5*). Furthermore, our investigation revealed that the inhibition of CRTAM expression led to increased early apoptosis in HFLS cells, as demonstrated through Annexin V/PI staining (*Supplementary 5*). Remarkably, the invasive capacity of HFLS cells was significantly diminished with the use of si-CRTAM compared to siNC (*Supplementary 5*). Additionally, our research uncovered a negative correlation between the expression level of CRTAM and the formation of cell colonies in HFLS cells (*Supplementary 5*). These noteworthy findings strongly suggest that CRTAM plays a crucial role in facilitating the proliferation and invasion capabilities of RA.

Next, we performed a correlation analysis of CRTAM in RA transcriptome, and the detailed correlation index was summarized in *Supplementary 6*. We found that CRTAM might regulate T cell activation, cytokine receptor activity in GO term, and fatty acid degradation, pyruvate metabolism in KEGG; S—acyltransferase, L—amino acid transmembrane transporter activity in GSEA analysis (*Supplementary 7*). We also found that *CRTAM* displayed a significantly negative correlation with *PTTG1IP* and a positive correlation with *ITGB2* and *MMP13* (Figure 5(b)). In addition, *MMP13*, *ITGB2*, and *CRTAM* were positively correlated with ESTIMATE, immune score, and stromal score (Figure 5(c)). We further verified this phenomenon in two independent correlation analyses. *ITGB2* was significantly related to the antigen processing machinery (APM) and CD8 T effector signature (Figure 5(d)). Such a relationship was found in Figure 5(e), since *ITGB2* was positively correlated with nearly all immune cell signatures, and the highest correlation index was found in effector memory CD8 T cells, MDSCs, and regulatory T cells.

### 3.5. Construction and Verification of the RA-Related Risk Model

**Figure 6:**
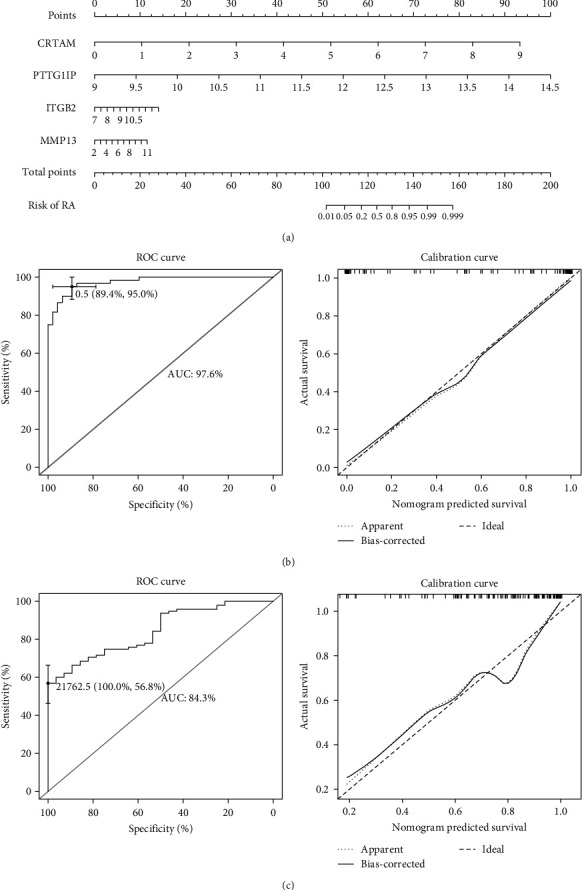
Construction and verification of the susceptibility quantification system for RA. (a) Nomogram based on the expression of four biomarkers to predict the susceptibility scores of RA arthritis patients. (b, c) ROA curve and calibration curve of the prediction system in the training and testing cohorts.

Based on the expression level and coefficients of four signatures, we constructed a novel RA-related risk score prediction model based on the training dataset (Figure 6(a)). The RA risk score was calculated by summing the scores of the gene expressions multiplied by the corresponding coefficients. The ROC value of the training dataset reached 0.976, which was proven by the calibration curve, which was nearly identical to the ideal model (Figure 6(b)). In addition, such high accuracy and sensitivity were detected in the test cohort from GSE89408, which also displayed a high AUC value (0.843) and satisfactory calibration curve (Figure 6(c)).

### 3.6. Two Distinctive RA Subtypes Led to Distinctive Immune Phenotypes

**Figure 7:**
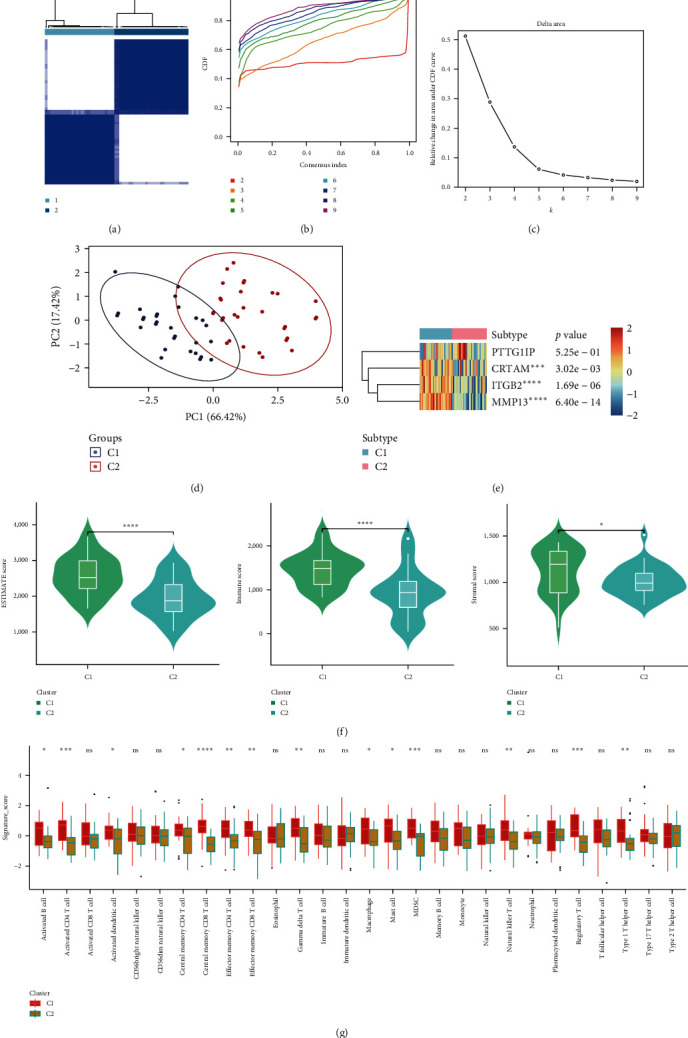
Identification of two distinctive subtypes in the RA groups. (a) Consensus cluster matrix of RA patients when *k* turns to 2. (b) The cumulative distribution function curves suggested *k*2 as the optimal cluster number in RA patients. (c) The relative change in area under the CDF curve. (d) 2D principal component plot by the matrix derived from the four signatures. The blue dots represent C1, and the red dots represent C2. (e) Heatmap illustrating the different expression levels of four biomarkers between C1 and C2. (f) Differences in ESTIMATE score, immune score, and stromal score between C1 and C2. (g) Difference in immune infiltration score between C1 and C2.  ^*∗*^*P* < 0.05,  ^*∗∗*^*P* < 0.01,  ^*∗∗∗*^*P* < 0.001,  ^*∗∗∗∗*^*P*-value is too small, close to zero.

In this part, we divided RA samples into two distinctive subtypes based on the *CRTAM*, *PTTG1IP*, *ITGB2*, and *MMP13* expression matrix (Figure 7(a)). The optimal cluster number was determined through consensus CDF and relative changes in regions under the CDF curve (Figures 7(b) and 7(c)). The PCA plot further showed the heterogeneity between subtypes (Figure 7(d)).  Figure 7(e) reveals the significantly different expression levels of *CRTAM*, *ITGB2*, and *MMP13*, which also reinforces the significant role of the three signatures in explaining the heterogeneity of RA samples. We also found an immune-activated state of C1 among RA samples, since ESTIMATE, immune score, and stromal score were higher in this subtype (Figure 7(f)). Most immune cell infiltration scores were also higher in the C1 subtype (Figure 7(g)). Combined with the higher expression of *CRTAM*, *ITGB2*, and *MMP13* in C1, we presumed that the three signatures could exacerbate RA immune dysregulation and be involved in RA progression.

### 3.7. Impact of CRTAM across Various Cancers

**Figure 8:**
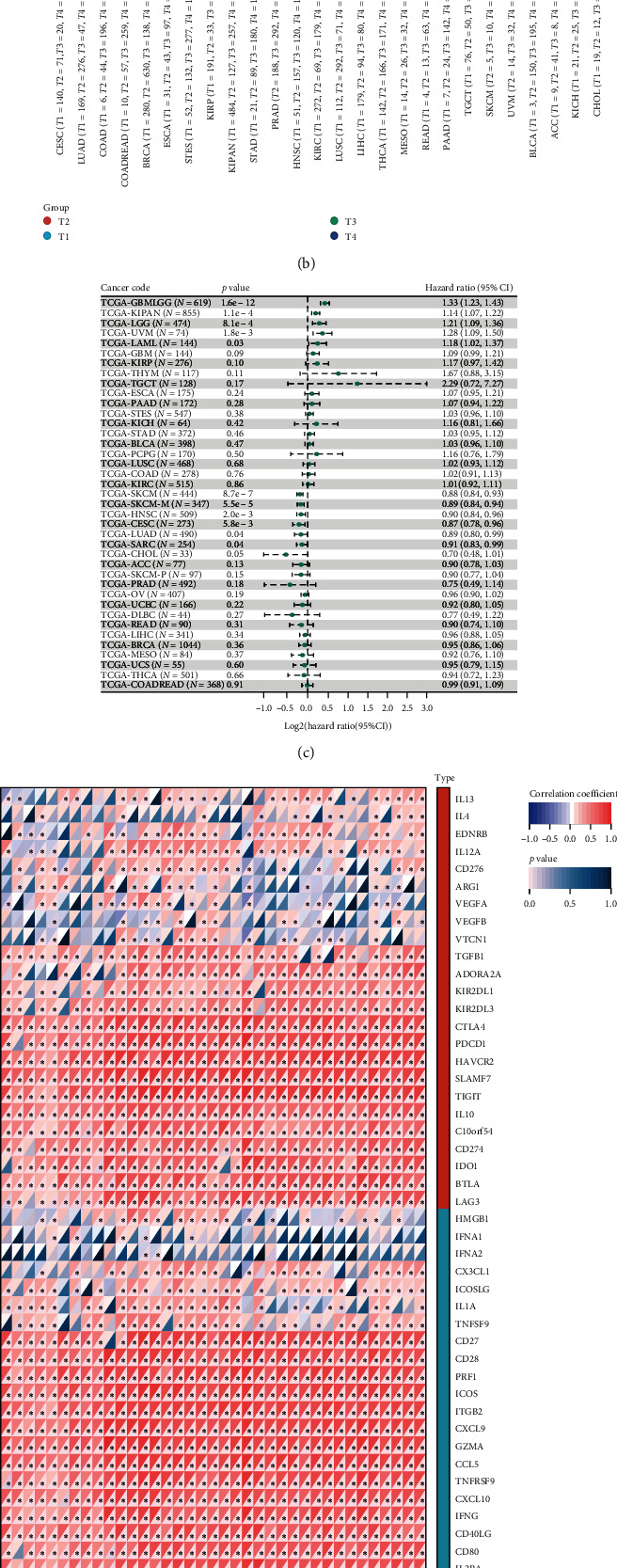
Characteristics of CRTAM across cancers. (a) Expression level of *CRTAM* between cancer and normal tissues. (b) Differential expression of *CRTAM* in different stages of pan cancer. (c) Univariable Cox analysis of *CRTAM* on overall survival. (d) Correlation of *CRTAM* and immune-related signatures.  ^*∗*^*P* < 0.05,  ^*∗∗*^*P* < 0.01,  ^*∗∗∗∗*^*P*-value is too small, close to zero.

**Figure 9:**
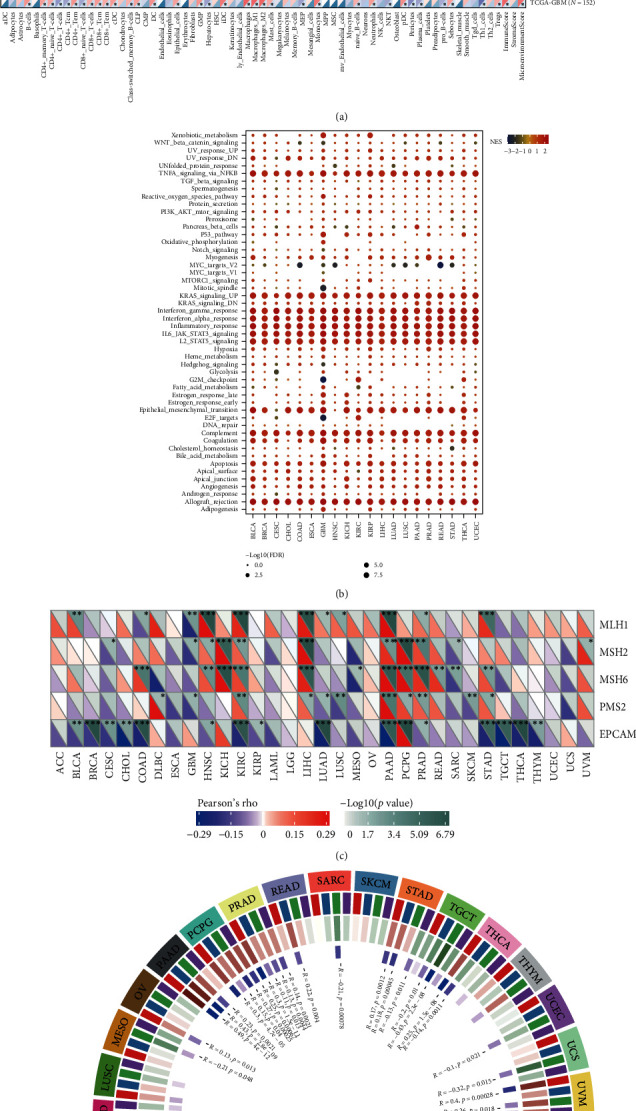
Biological influence of CRTAM across cancers. (a) Correlation of *CRTAM* and immune infiltration based on the Xcell algorithm. (b) GSEA results based on high *CRTAM* expression group vs. low *CRTAM* expression group. (c, d) Correlations of *CRTAM* expression with the expression levels of MMR and DNA methyltransferase signatures.  ^*∗*^*P* < 0.05,  ^*∗∗*^*P* < 0.01,  ^*∗∗∗*^*P* < 0.001.

Since several studies found that the consistent immune dysregulated state was not only involved in RA but also affected cancer patients, we further decided to link the relationship of *CRTAM* across cancers based on its high AUC value in both the training and testing cohorts.  Figure 8(a) shows that *CRTAM* was more highly expressed in tumor tissues among BRCA, CESC, ESCA, STES, KIRP, KIPAN, COAD, READ, STAD, HNSC, KIRC, SKCM, THCA, OV, PAAD, TGCT and LAML, while it displayed the opposite trend among GBM, LGG, LUAD, LUSC, WT, READ, and ALL. In addition, the *CRTAM* expression level was higher in advanced stage samples across STES, KIPAN, STAD, PRAD, KIRC, READ, and BLCA, while it was more highly expressed in early-stage samples among LUAD, HNSC and THCA (Figure 8(b)). Through prognostic analysis, we found that CRTAM could affect OS among GBM/LGG, KIRAN, LGG, UVM, and LAML, PFI among GBM/LGG, KIRAN, LGG, CESC, ACC, SKCM, HNSC, and CHOL, DSS among GBM/LGG, KIRAN, LGG, UVM, SKCM, CESC, HNSC and PRAD, and DFI in KIRP (Figure 8(c), *Supplementary 8*). Regarding the immune impact of *CRTAM* in cancers, we surprisingly found that *CRTAM* was positively related to nearly all immune-related modulators, including chemokines, chemokine receptors, MHCs, immune inhibitors, and immune stimulators (Figure 8(d)). Through the Xcell algorithm, we found a paradigmatic positive correlation between *CRTAM* and aDC, CD8 naïve T cells, CD8 T cells, CD8 Tems, cDCs, DCs, macrophages, M1 macrophages, M2 macrophages, monocytes, pDCs, immune score and microenvironment score, and a negative relationship with MEPs and osteoblasts among all cancers (Figure 9(a)). *CRTAM* could paradigmatically activate *TNF*, *KRAS*, interferon, inflammatory, IL6-JAK-STAT3, IL2-STAT5, epithelial-mesenchymal transition, and complement-related signatures across cancers (Figure 9(b)). In addition, we also found that *CRTAM* displayed a significant correlation with DNA mismatch repair (MMR) signatures, including *MLH1*, *MSH2*, *MSH6*, *PMS2*, and *EPCAM*, among COAD, HNSC, KICH, KIRC, LIHC, PAAD, PCPG, PRAD, and STAD (Figure 9(c)). For DNA methylation catalyzed by DNMTs, we investigated the correlation between CRTAM expression and the expression of four essential DNMTs across cancers and found that CRTAM was significantly related to DNMTs in PAAD, PCPG, PRAD, STAD, TGCT, UVM, BLCA, COAD, KICH, KIRC, and LIHC (Figure 9(d)). All these findings reminded us that *CRTAM* might be involved in oncogenesis by influencing DNA methylation.

### 3.8. CTRAM Owned a Better Performance in Predicting RA than Classic Biomarkers

To investigating that whether *CRTAM* owned a better diagnostic efficacy than classic RA related biomarkers, we carried a systematic comparison analysis between *CTRAM* and those genes, including *MMP3*, *S100A8*, *S100A9*, *IL6*, *COMP*, *LAG3*, and *ENTPD1*. As shown in *Supplementary 9*, we found that the expression level of *CRTAM*, *MMP3*, *S100A8*, *S100A9*, and *ENTPD1* was higher in RA tissues in both training and testing cohorts. Surprisingly, *CTRAM* led the highest AUC value in both training and testing cohort (0.875 and 0.948, respectively) (*Supplementary 9*). To better understanding and comparing the distinctive biological roles of those signatures, we conducted a systematic correlation analysis in RA expression matrix. The results reminded us of that CTRAM was mainly involved in leukocyte proliferation, regulation of T cell activation, and osteoclast differentiation with *NECTIN2* and *CADM1* (*Supplementary 9* and *Supplementary 10*). According to correlation analysis, our findings were consistent with the other seven classic genes reported in previous works because of *MMP3* mainly involving in leukocyte migration and RA by interacting with *TIMP2*, *TIMP1*, and *MMP10* (*Supplementary 9* and *Supplementary 10*), *S100A8* and *S100A9* regulating leukocyte mediated immunity, phagosome and RA with *S100A12* and *CDC34* (*Supplementary 9* and *Supplementary 10*), *IL6* participating in cytokine mediated and IL − 17 signaling pathway with *IL6R* and *IL6ST* (*Supplementary 9* and *Supplementary 10*), *COMP* involving in focal adhesion by interacting with *MATN3*, *MATN1*, *MATN4*, *ADAMTS7*, and *ADAMS12* (*Supplementary 9* and *Supplementary 10*), *LAG3* activating cytokine mediated signaling pathway and Th1 and Th2 cell differentiation with *FGL1* and *CENPJ* (*Supplementary 9* and *Supplementary 10*), *ENTPD1* characterized with histone modification and Insulin signaling pathway in RA with ENTPD family members (*Supplementary 9* and *Supplementary 10*). All those results suggested us that CRTAM was a novel and independent biomarker for RA.

## 4. Discussion

RA is a systemic inflammatory autoimmune disease characterized by swelling and pain in multiple joints as well as symmetric polyarthritis [4, 27, 28]. Chronic joint deformities, disability, and increased mortality can result from untreated RA [29]. As life expectancy increases worldwide, the number of elderly people with RA is also rising, and young people are becoming more susceptible to the disease [2]. Thus, it is urgent to identify novel pathogenesis-related targets to prevent the progression of RA at an early stage [30]. In recent years, some novel molecular biomarkers have been identified for the diagnosis of RA [31–33]. However, those previous studies were based on small sample sizes, which might influence the reliability of their findings. Commonly, RA is notorious for sustained inflammation of the tendon, which leads to bone and cartilage destruction. Hormone level, family history, and carraraite consumption functioned as susceptibility factors for RA [34, 35]. All this accumulating evidence suggests that RA is a polygenic disease with multiple signatures evolving in its progression. Based on this hypothesis, the underlying mechanism of RA remains unclear and requires further investigation. Advances in sequencing technology followed by bioinformatics aided the reanalysis of previous datasets of RA to identify the mechanism and pathogenesis-related targets. Applying machine learning algorithms to biomedicine largely promotes a better understanding and deconvolution of high-sequence information. Several studies have tried to decipher the heterogeneity of RA to some extent and have made limited advances in better understanding RA [33, 36]. While the obsolete algorithm limited the reliability of clinical practice, a comprehensive understanding of RA in multiple cohorts with advanced machine learning algorithms is extremely urgent.

In this study, we identified and verified four biomarkers with the use of four machine learning algorithms for the prediction of RA susceptibility from six RA datasets containing nearly 300 samples. We first constructed integrated datasets derived from five RA datasets from the GEO platform after batch effect removal. We then screened out DEGs between RA and normal tissues, and the DEGs were annotated into several novel immune-related signatures in addition to the classic RA pathway, such as the activation of chemokines, Th1, 2, 17 cell differentiation, and inhibition of tyrosine metabolism, insulin, calcium, and AMPK signaling pathways. Part of these findings were consistent with previous works. Since RA is characterized by an imbalance of Tregs and Th17s, numerous studies have suggested that targeting Th cell subtypes could alleviate RA progression. Ye et al. [37] utilized the CK2 inhibitor CX4945 to inhibit Th1 and Th17 cell responses while promoting Th2 cell responses in RA, which significantly dampened IFN-*γ* and IL-17A production and alleviated the inflammatory state. Rao et al. [38] identified an expanded population of PD-1^hi^ CXCR5- “peripheral helper T (TPH) cells that express factors that enable the activation of B cells, including IL-21, CXCL13, ICOS, and MAF, with the application of multidimensional cytometry, transcriptomics, and functional assays”. Traditional disease-modifying antirheumatic drugs (DMARDs), including methotrexate (MTX) and leflunomide (LEF), are the mainstay of RA symptomatic treatment, the purpose of which is to reduce inflammation and prevent the progression of disease. As DMARDs have been extensively used in worldwide clinical treatment, drug resistance has also become a problem, and new therapies are urgently needed. On the other hand, we found that several metabolism-related pathways were inhibited in RA, which could be treated as novel therapeutic approaches with specific agonists. Previous studies have indicated that targeting tyrosine kinase-related pathways could alleviate RA patients' symptoms and decrease the adverse effect of immune-mediated disorders, e.g., SLE [39, 40]. Numerous studies have also found that the inhibited state of the energy sensor AMPK could aggravate mitochondrial insufficiency, thus enhancing the early stages of tolerance breakdown and the late stages of tissue inflammation in RA. In addition, we first found in our work that activating insulin- and calcium-related pathways could be a new weapon for treating RA.

In addition, a risk score nomogram constructed and tested based on four signatures was able to distinguish RA from normal tissues. The AUC values of the training and test cohorts were both greater than 0.8, which revealed the high accuracy of our model. It should be mentioned that several studies have reported the association between OA and RA [41]. The nomogram constructed based on RA-related signatures could also perform well in OA patients. Several signatures of the four genes have been reported previously. Yang et al. [42] reported several hub genes involved in RA containing *ITGB2*, which had the highest diagnostic value and higher expression in RA compared with OA. Nearly all extracellular matrix components are degraded in RA by MMPs induced by inflammatory cytokines such as IL-1*β* and TNF-*α*. Due to their role as rate-limiting enzymes in collagen degradation, *MMP1* and *MMP13* collagenases play an important role in RA [43, 44]. *MMP1* is produced by synovial cells, while *MMP13* is produced by chondrocytes in cartilage [45]. Our study further verified the diagnostic value of *MMP13* in RA in addition to its therapeutic potential. A study from Chen et al. [46] found that overexpression of *circ-PTTG1IP* was detected in RA patients and RA-FLSs, and knockdown of circ-*PTTG1IP* suppressed cell proliferation, migration, invasion, and inflammation. The results from those studies proved the reliability of our findings. Meanwhile, it should be mentioned that the diagnostic value of *CRTAM* in RA was first investigated in this work, and the exact biological role of *CRTAM* in RA patients still warrants further studies.

To investigate the role of the four signatures in RA immune infiltration, we also conducted a comprehensive correlation analysis between genes and pathways. Except for *PTTG1IP*, *MMP13*, *ITGB2*, and *CRTAM* were strongly related to immune, stromal score, and immune infiltration degree. The higher expression levels of the three genes in RA tissues and the positive correlation coefficient reminded us that *MMP13*, *ITGB2*, and *CRTAM* might be involved in the progression of RA by activating abnormal immune infiltration. *ITGB2 (CD18*), as an integrin subunit, is a heterodimeric surface receptor expressed specifically by leukocytes. In general, *ITGB2* is involved in the development, metastasis, and invasion of a wide range of tumor types, including liver cancer, colon cancer, breast cancer, and leukemia [47–49]. Xu et al. [50] revealed that the expression of *ITGB2* stratifies glioma patients into high and low subgroups, with different clinical outcomes and immune activation states; a higher level of *ITGB2* expression in glioma patients was associated with a better immune response, which was consistent with our findings that the *ITGB1* expression level positively correlated with immune infiltration in RA. Philllips [45] found that blockade of *MMP13* alleviated posttraumatic osteoarthritis through inhibition of immune restructuring, angiogenesis, innate immune response, and proteolysis. Our study emphasized its role in immune cell recruitment in RA tissues and its high sensitivity to predict RA susceptibility.

Previous studies found that RA was correlated with various cancers. There is evidence that RA increases the risk of cancer, including lung cancer, lymphoma, and breast cancer [51–53]. Meanwhile, immunosuppressive agents used to treat RA have been shown to increase cardiovascular disease and cancer risk factors [54]. Thus, we further investigated *CRTAM*'s role in pan cancer since the ROC values of *CRTAM* displayed the highest score in both the training and test cohorts. *CRTAM*, a cytotoxic and regulatory T-cell molecule that encodes a type I transmembrane protein with Ig domains such as V and C1 in CD4-positive and CD8-positive T cells, controls T-cell activation and differentiation, as well as tissue retention, by mediating heterophilic cell‒cell adhesion. We thus hypothesized that consistently elevated expression of *CRTAM* in RA patients could increase the probability of several cancer types. Through pan cancer analysis, we found that *CRTAM* was more highly expressed and functioned as a hazardous factor of prognosis in GBM, LGG, KIPAN, UVM, and LAML. Kuo et al. [55] performed a genetic association study and found that three loci, including *CRTAM*, could increase the susceptibility of nonhuman papillomavirus (HPV)-driven oropharyngeal cancer. Moreover, *CRTAM* expression levels were strongly correlated with chemokine receptor, immune inhibitor, DC, CD8 Tcm cell, Tem cell, macrophage, and Treg cell levels across cancers, which was consistent with the enrichment analysis in this work. According to our prior results, these high infiltration fractions of cells are consistent with the *CRTAM* we observed in RA. These high correlation coefficients of *CRTAM* among cancers and RA tissues largely reminded us of that *CRTAM* could play a paradigmatic role in immune infiltration.

To the best of our knowledge, this work is the largest sample-based and multialgorithm study in RA. Even our study made some advances and novel findings in RA, which might help a better understanding of RA and cancers. There are still some limitations as follows. First, most of our work was based on bioinformatics, and even though we collected tissues to verify the different expression levels of the four novel biomarkers, the sensitivity and specificity of those genes need to be explored in larger prospective studies. Second, nearly all datasets enrolled in our work belong to Western countries, and whether the findings are applicable to other ethnic groups remains unknown. Lastly, we have investigated the potential biological roles by association analysis, while the detailed influence of *CRTAM* on RA and tumors requires more experiments for validation.

## 5. Conclusion

In general, this study applied machine learning algorithms to large sample-based RA cohorts to identify specific hub signatures between RA and normal tissue. Four promising biomarkers, *CRTAM*, *PTTG1IP*, *MMP13*, and *ITGB2*, were found and verified. Targeting metabolic pathways, especially activating tyrosine metabolism and the insulin, calcium, and AMPK signaling pathways, was first proposed in our work. We also connected the potential impact of *CRTAM* in various cancers, which broadened researchers' understanding of RA and carcinoma.

## Data Availability

The datasets adopted in this study can be found in the Materials and Methods section.

## Supplementary Materials

Supplementary 1 Summary of baseline information of six dataset enrolled in this work.

Supplementary 2 Deconvolution file for ssGSEA analysis.

Supplementary 3 Enrichment analysis results of (a) cellular components and (b) molecular functions of DEGs between RA and normal tissues.

Supplementary 4 ROC curve and different expression levels of four signatures in distinguishing OA from normal tissues in (a) GSE1919, (b) GSE12021, (c) GSE55235, (d) GSE55457, and (e) GSE82107.

Supplementary 5 Validation of the different expression levels of four novel signatures from patient tissues and detecting the role of *CRTAM* in RA.

Supplementary 6 Correlation of CRTAM with remained mRNA in RA tissues.

Supplementary 7 Biological roles of CRTAM in RA based on guilt-of-association analysis.

Supplementary 8 (a–c) Univariable Cox analysis of *CRTAM* on PFI, DSS, and DFI.

Supplementary 9 (a) Different expression level of eight biomarkers in both training (up) and testing (down) cohort. (b) Comparison of diagnostic efficacy of eight biomarkers in training and testing datasets (c). GO term (left) and KEGG pathway (right) analysis of *CRTAM* (d), *MMP3* (e), *S100A8* (f), *S100A9* (g), *IL6* (h), *COMP* (i), *LAG3* (j), and *ENTPD1* (k) in RA samples.

Supplementary 10 (a) Protein interaction network of *CRTAM*, *MMP3* (b), *S100A8* (c), *S100A9* (c), *IL6* (d), *COMP* (e), *LAG3* (f), and *ENTPD1* (g) based on datasets from genemania website.
