# Supplementary figures and images for: Identification and Verification of Novel Biomarkers Involving Rheumatoid Arthritis with Multimachine Learning Algorithms: An In Silicon and In Vivo Study
Source: Mediators Inflamm. 2024 Feb 14;2024:3188216. doi: 10.1155/2024/3188216 (PMC10881253; doi:10.1155/2024/3188216)

Figure S1

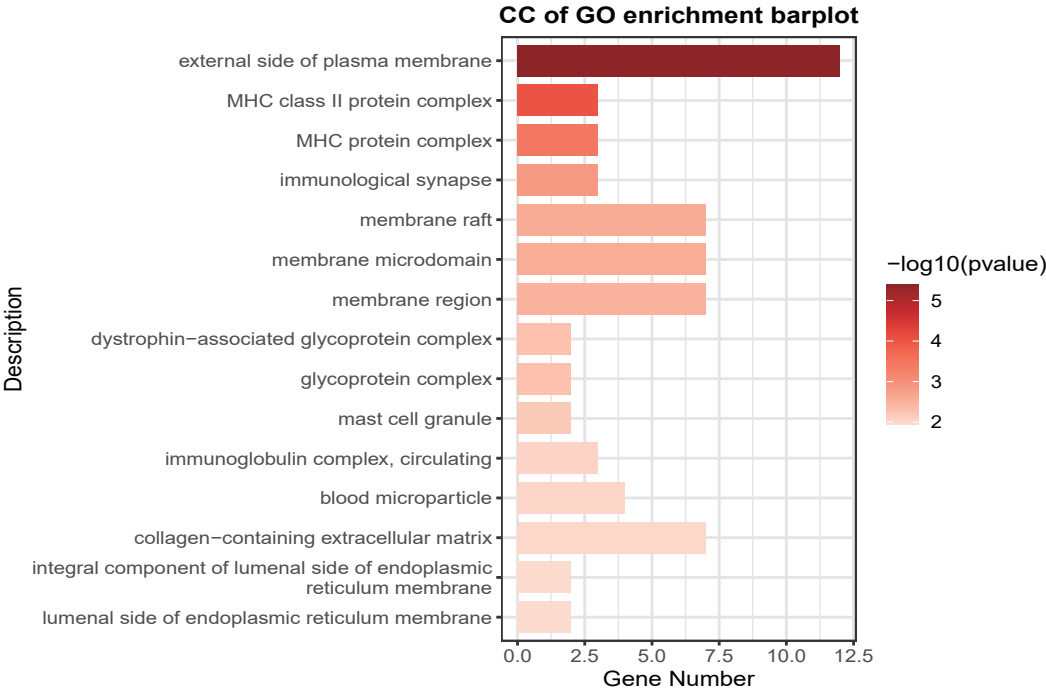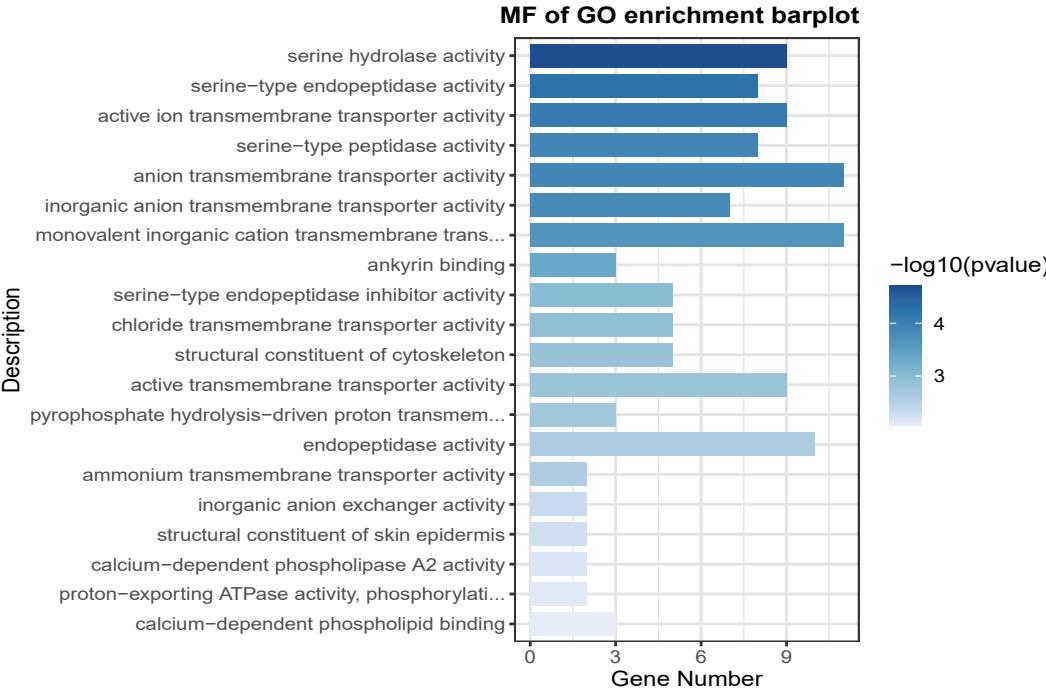

Supplement: Supplementary 3 — Enrichment analysis results of (a) cellular components and (b) molecular functions of DEGs between RA and normal tissues. [file 3188216.f3.pdf]

Figure S3

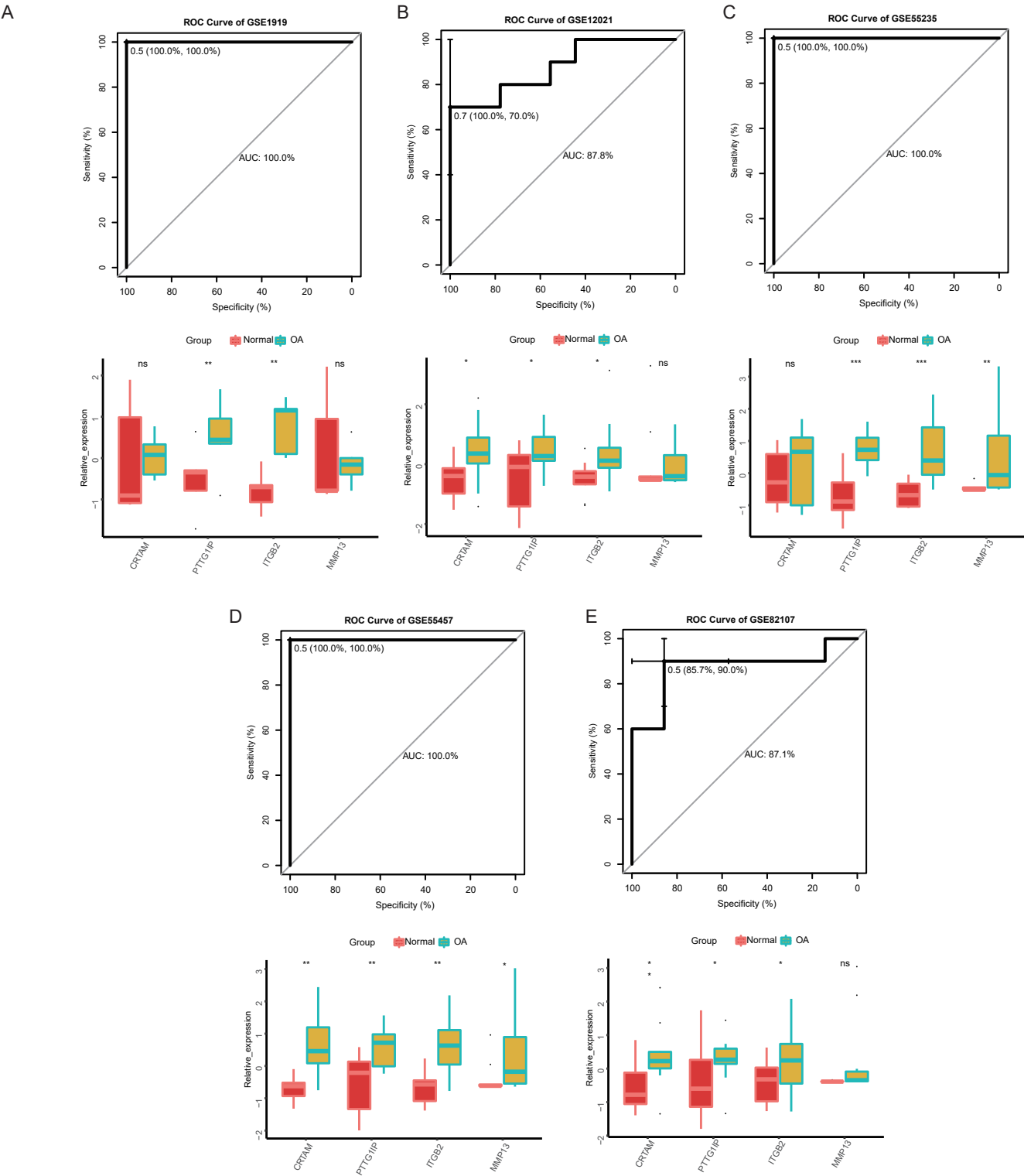

Supplement: Supplementary 4 — ROC curve and different expression levels of four signatures in distinguishing OA from normal tissues in (a) GSE1919, (b) GSE12021, (c) GSE55235, (d) GSE55457, and (e) GSE82107. [file 3188216.f4.pdf]

Figure S2

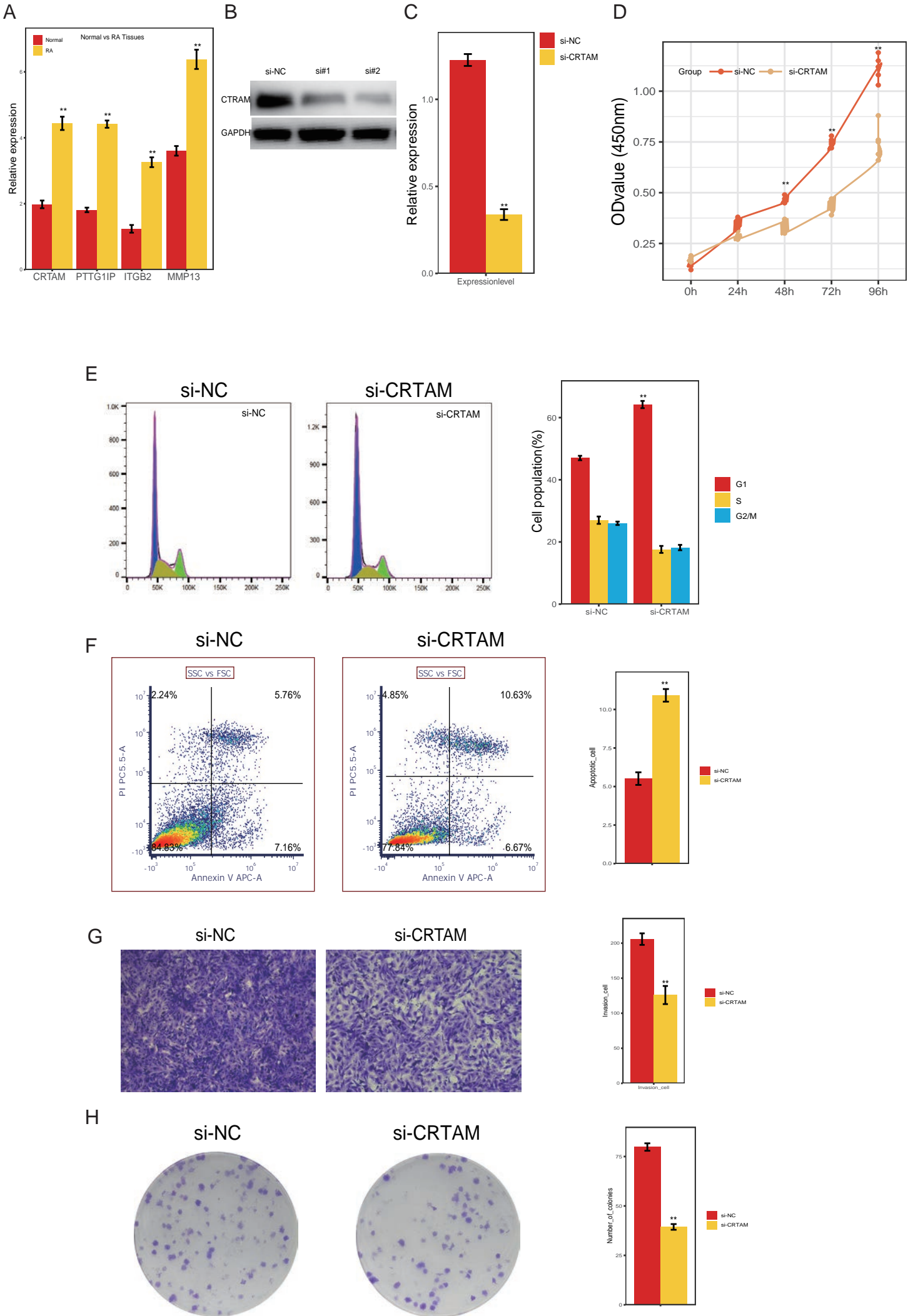

Supplement: Supplementary 5 — Validation of the different expression levels of four novel signatures from patient tissues and detecting the role of CRTAM in RA. [file 3188216.f5.pdf]

Figure S4

A

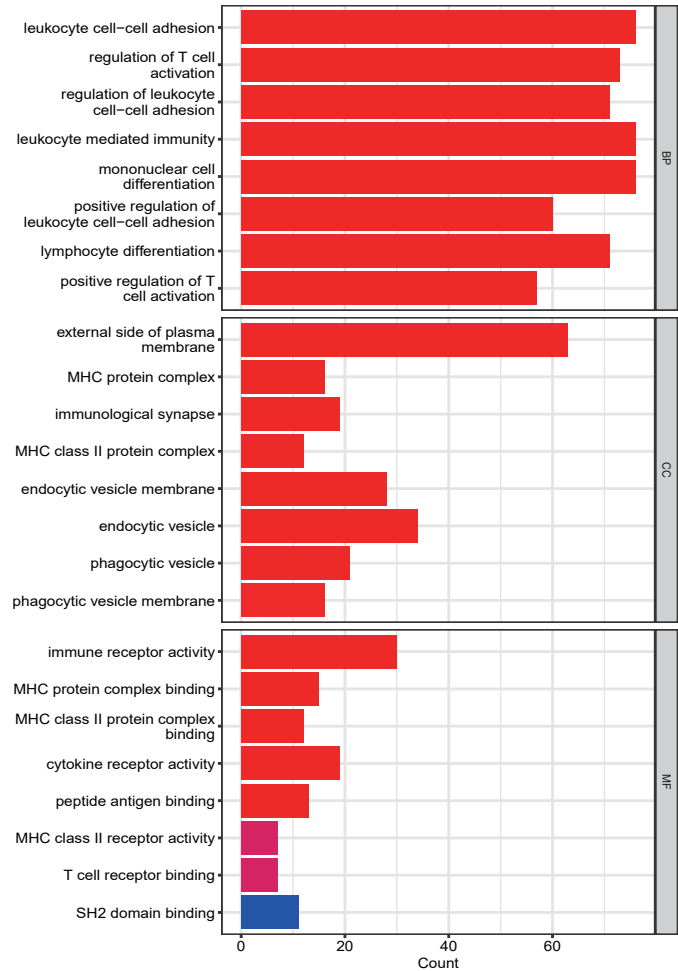

B

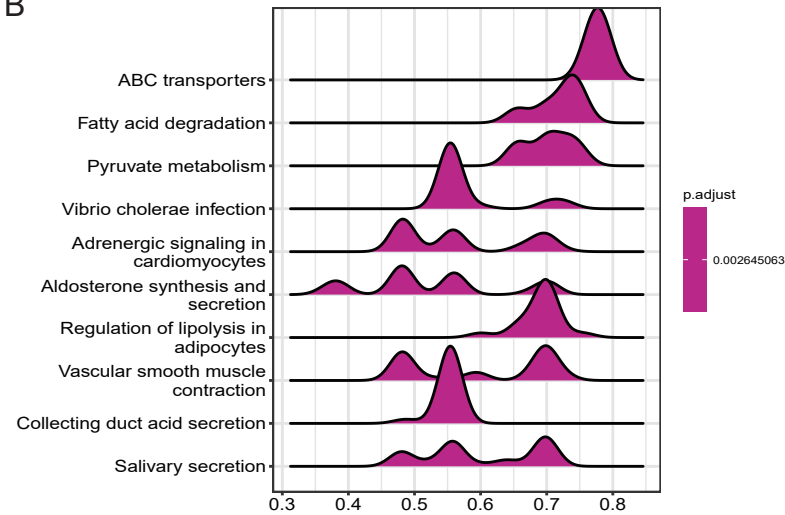

C

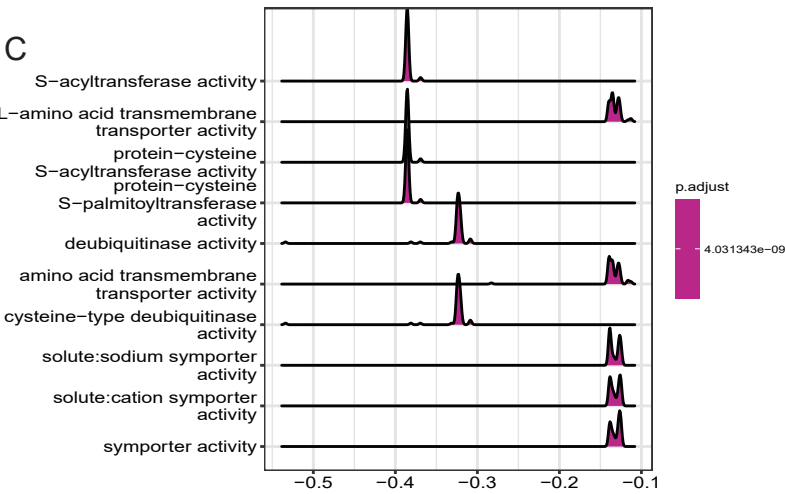

Supplement: Supplementary 7 — Biological roles of CRTAM in RA based on guilt-of-association analysis. [file 3188216.f7.pdf]

Figure S5

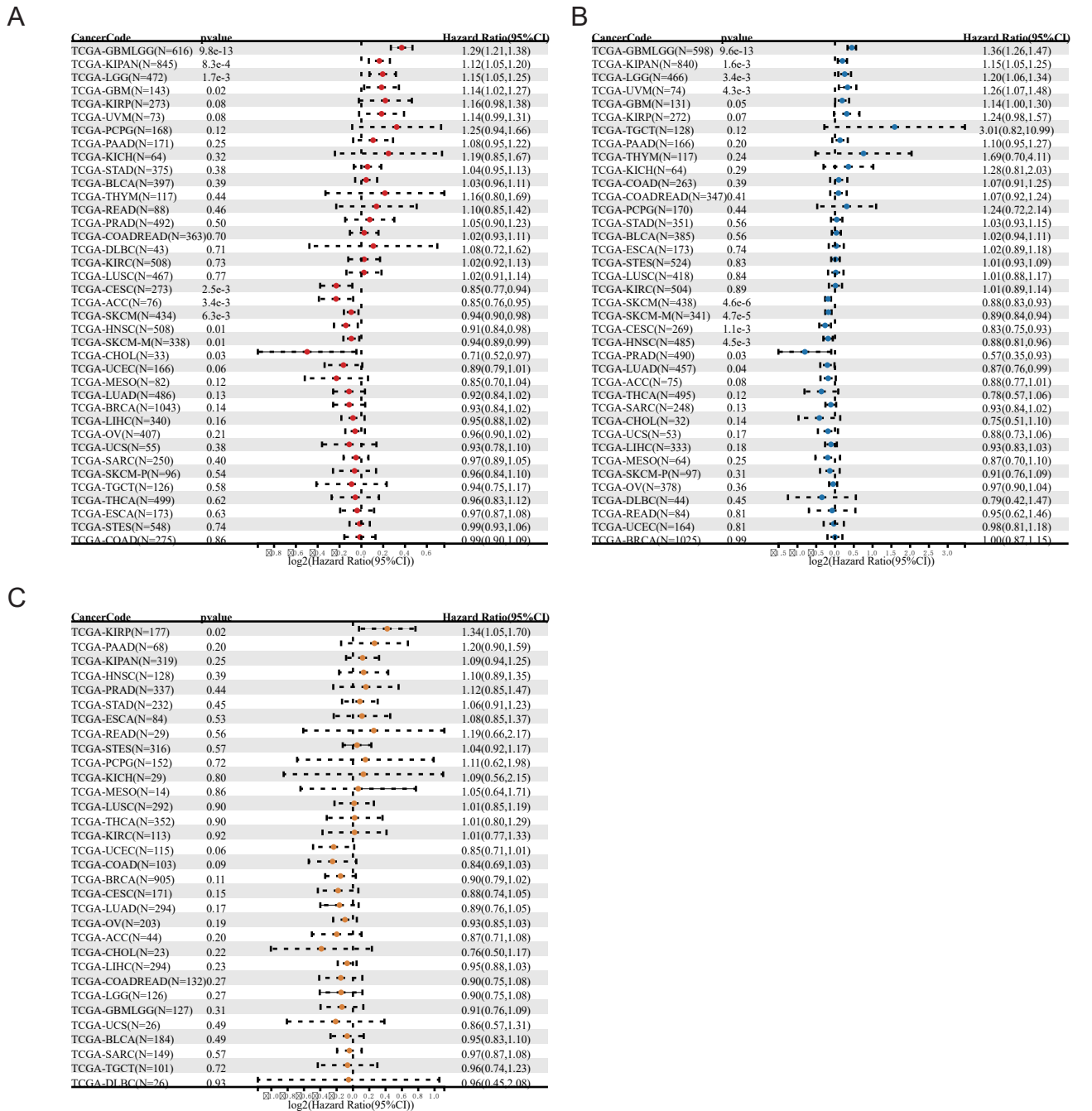

Supplement: Supplementary 8 — (a–c) Univariable Cox analysis of CRTAM on PFI, DSS, and DFI. [file 3188216.f8.pdf]

Figure S6

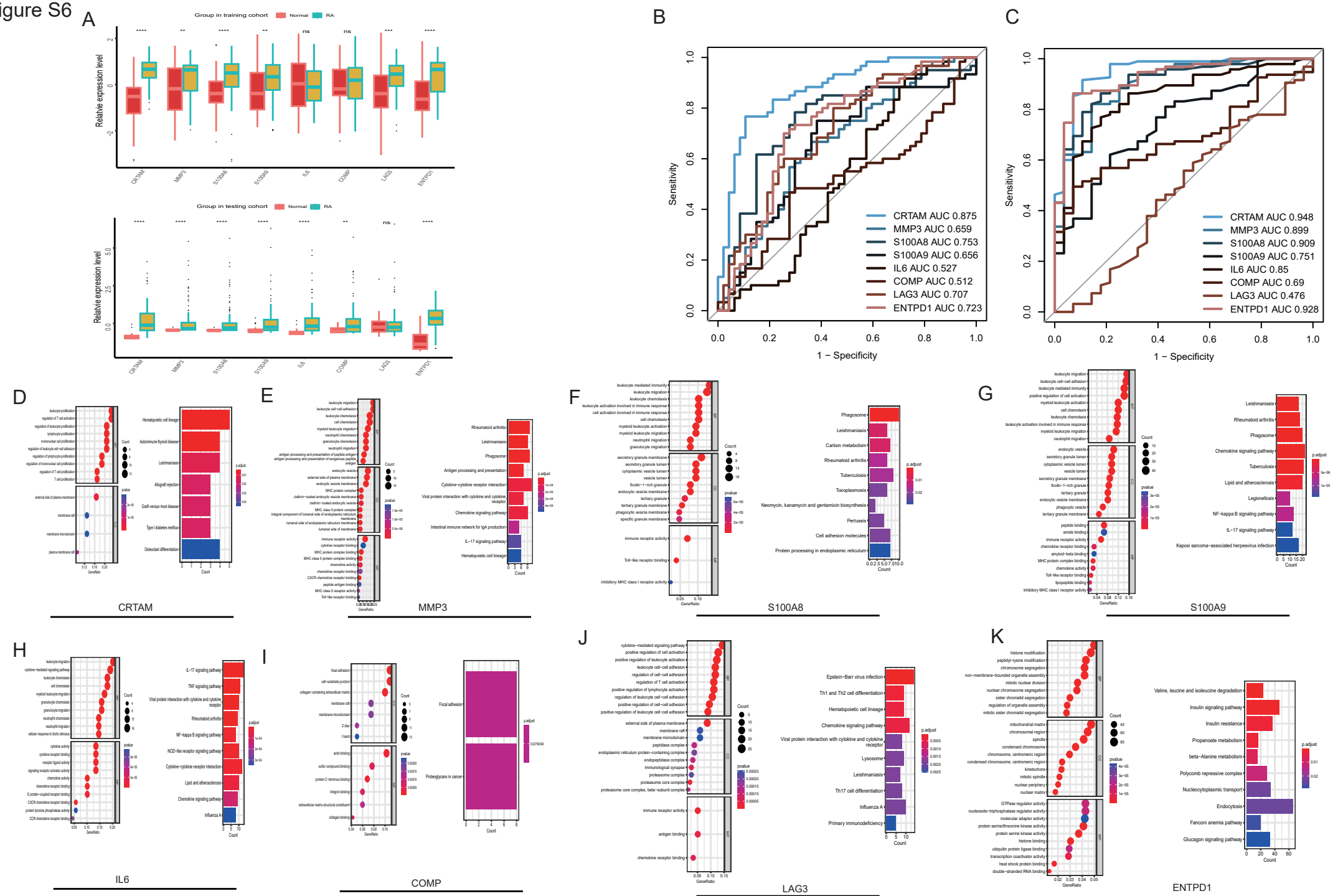

Supplement: Supplementary 9 — (a) Different expression level of eight biomarkers in both training (up) and testing (down) cohort. (b) Comparison of diagnostic efficacy of eight biomarkers in training and testing datasets (c). GO term (left) and KEGG pathway (right) analysis of CRTAM (d), MMP3 (e), S100A8 (f), S100A9 (g), IL6 (h), COMP (i), LAG3 (j), and ENTPD1 (k) in RA samples. [file 3188216.f9.pdf]

Figure S7

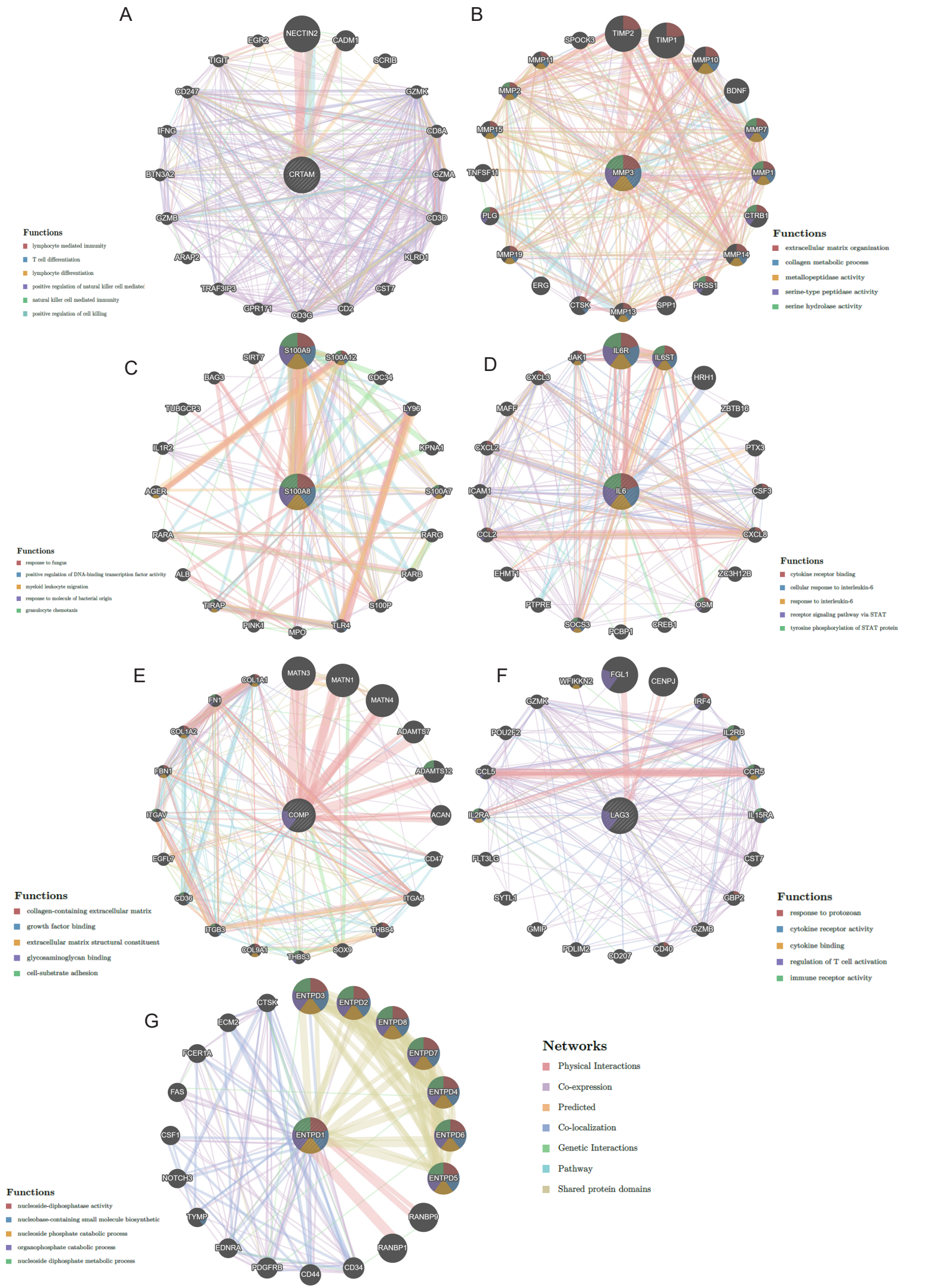

Supplement: Supplementary 10 — (a) Protein interaction network of CRTAM, MMP3 (b), S100A8 (c), S100A9 (c), IL6 (d), COMP (e), LAG3 (f), and ENTPD1 (g) based on datasets from genemania website. [file 3188216.f10.pdf]
